# Application of the Portuguese version of the Obstructive Sleep Apnea-18 survey to children

**DOI:** 10.5935/1808-8694.20130132

**Published:** 2015-10-08

**Authors:** Fausto Manuel Vigario Santos Fernandes, Rafaela da Cruz Vieira Veloso Teles

**Affiliations:** a Head of the ENT Service (Head of the Hospital ENT Service); b Bachelor's Degree in Medicine (ENT Intern, Alto Ave Hospital Center, Guimarães). Otorhinolaryngology Service of the Alto Ave. Hospital Center

**Keywords:** child development, health care surveys, sleep apnea, obstructive

## Abstract

Despite the significant prevalence of obstructive sleep apnea syndrome (OSAS) in children, the diagnosis and treatment of this condition is still challenging due to the difficulties inherent to objectively assessing the disease's severity.

**Objective:**

To verify whether the Portuguese version of the Obstructive Sleep Apnea-18 (OSA-18) survey is as valid as its original version in English.

**Method:**

Prospective study. The OSA-18 was translated into Portuguese, culturally adapted, and tested in the Portuguese population. The caregivers of 51 children (aged from 2 to 12 years) diagnosed with OSAS answered the OSA-18-pv survey. Statistical analysis was used to assess the psychometric properties of the survey.

**Results:**

Reliability analysis yielded a Cronbach's alpha of 0.821, confirming the survey's consistency. Converging validity was assessed using Pearson's correlation coefficient, which revealed a statistically significant correlation between individual data and total results. The survey can be easily and quickly answered (7.26 min). The outcomes of the OSA-18-pv showed that quality of life was poor in 14 children with OSAS (28%), moderate in 22 (43%), and high in 15 (29%).

**Conclusion:**

the OSA-18-pv is a valid tool and can be used to assess the impact on the quality of life of Portuguese children with OSAS.

## INTRODUCTION

Obstructive sleep apnea syndrome (OSAS) is a respiratory disorder characterized by prolonged partial obstruction and/or intermittent complete obstruction of the upper airways and interruption of normal ventilation during sleep^1^.

Pediatric OSAS became an important topic not only because of its high prevalence, but also due to its associations with different comorbidities, some of which bearing possible implications to the affected subject's adult life. Moreover, it has been established that the pathophysiology of pediatric OSAS is different from that of OSAS in adult subjects. Although its prevalence is yet unknown, pediatric OSAS has been estimated to affect 1%-2 % of children, with cases peaking between the ages of two and eight years, at a time in which the Waldeyer's ring is enlarged^2^.

OSAS is the main indication for tonsillectomy and adenoidectomy in children. In the last two decades, a progressive decrease was observed in the number of adenotonsillectomy procedures for recurrent infections and an increase in the number of such procedures for OSAS^3^. Surgery has been proven effective in controlling neurocognitive sequelae of OSAS, such as poor school performance and attention deficit and hyperactivity disorder, in improving left and right ventricular ejection fraction, and in decreasing the levels of biomarkers of inflammation^4^. However, pediatric OSAS remains underdiagnosed^5^ and, therefore, undertreated. Polysomnography (PSG) is currently the gold standard test for the diagnosis of OSAS in children. However, PSG is expensive, time consuming, and not all sleep labs run this test in children. The correct diagnosis and the decision of when to surgically intervene is made difficult due to the limited availability of objective measures to determine the severity of OSAS.

Recently, Franco et al.^6^ developed a survey to assess the impact of OSAS in children and their caregivers based on 18 questions. This instrument, called the OSA-18 survey, inquires caregivers in five domains: sleep disturbance, physical symptoms, emotional symptoms, daytime function, and caregiver concerns; each item is given a score from one to seven, in which one means ‘never’ and seven means ‘always’.

The OSA-18 survey proved to be simple and quick to complete, and can be used in clinical or research settings. Its validity for the English-speaking population has been established.

This study aimed to translate, culturally adapt, and validate the Portuguese version of the OSA-18 (OSA-18-pv).

## METHOD

### Adaptation and translation into Portuguese

The original version of the OSA-18 survey (Annex 1) was translated by two bilingual physicians into Portuguese, translated back into English, so that then the final version in Portuguese were produced.

**Annex 1:** OSA-18 for children with OSAS - English version [4].

OSAS Quality of Life Survey (OSA-18) Date:__/___/___							
Name:___________________________							
For each question below, please circle the number that best describes how often each symptom or problem has occurred during the past 4 weeks. Please circle only one number per question. Thank you.							
	None of the time	Rarely any of the time	A little of the time	Some of the time	A good bit of the time	Most of the time	All of the time
Sleep Disturbance							
During the past 4 weeks, how often has your child had… … loud snoring?	1	2	3	4	5	6	7
… breath holding spells or pauses in breathing at night?	1	2	3	4	5	6	7
… choking or made gasping sound while asleep?	1	2	3	4	5	6	7
… restless sleep or frequent awakenings from sleep?	1	2	3	4	5	6	7
Physical Symptoms During the past 4 weeks, how often has your child had.							
… mouth breathing because of nasal obstruction?	1	2	3	4	5	6	7
… frequent colds or upper respiratory infections?	1	2	3	4	5	6	7
… nasal discharge or a runny nose?	1	2	3	4	5	6	7
… difficulty in swallowing foods?	1	2	3	4	5	6	7
Emotional Symptoms During the past 4 weeks, how often has your child had…							
… mood swings or temper tantrums?	1	2	3	4	5	6	7
… aggressive or hyperactive behavior?	1	2	3	4	5	6	7
… discipline problems?	1	2	3	4	5	6	7
Daytime Function During the past 4 weeks, how often has your child had…							
… excessive daytime sleepiness?	1	2	3	4	5	6	7
… aggressive or hyperactive behavior?	1	2	3	4	5	6	7
… discipline problems?	1	2	3	4	5	6	7
Caregiver Concerns							
During the past 4 weeks, how often have the problems described above…							
… caused you to worry about your child's general health?	1	2	3	4	5	6	7
… created concern that your child is not getting enough air?	1	2	3	4	5	6	7
… interfered with your ability to perform daily activities?	1	2	3	4	5	6	7
… made you frustrated?	1	2	3	4	5	6	7

### Sample and procedures

This study was carried out between March 2011 and March 2012, with patients seen at the Alto Ave Hospital Center in Guimarães, Portugal, for obstructive sleep apnea syndrome. The protocol was approved by the Ethics Committee of the institution. The following enrollment criteria were applied: (1) patients had to be aged between two and 12 years; (2) subjects had to have a history of sleep-disordered breathing suggestive of OSAS for a period exceeding three months. The exclusion criteria were as follows: (1) presence of craniofacial dysmorphic features; (2) patients with psychiatric disorders (except for attention deficit and hyperactivity disorder); or (3) neuromuscular diseases. The questionnaires were completed by the caregivers of children with a physician present in the room as they visited the hospital. A pretest with 10 questionnaires was conducted to assess the difficulties that the survey could present and whether the respondents had properly understood the questions. Some corrections were made considering the Portuguese socioeconomic and cultural context. The final version of the OSA-18 (Annex 2) was therefore applied to the first 51 caregivers of children meeting the enrollment criteria and diagnosed with OSAS.

**Annex 2:** Portuguese version of the OSA-18 (OSA-18-pv).

OSA-18 Versão Portuguesa (OSA-18-pv) Data:___/____/___							
Nome:_______________							
Em cada uma das questões seguintes, faça por favor um círculo àvolta do número que melhor descreve a frequência de cada sintoma ou problema nas últimas 4 semanas. Assinala apenas um número por questão. Obrigado.							
	Nunca	Quase Nunca	Poucas Vezes	Algumas Vezes	Bastantes vezes	Quase Sempre	Sempre
Distúrbio do Sono							
Nas últimas 4 semanas, com que frequência o seu filho teve…							
… ressonar alto?	1	2	3	4	5	6	7
… paragens na respiração durante a noite?	1	2	3	4	5	6	7
… engasgos ou respiração ofegante enquanto dormia?	1	2	3	4	5	6	7
… sono agitado ou despertares frequentes do sono?	1	2	3	4	5	6	7
Sintomas Físicos							
Nas últimas 4 semanas, com que frequência o seu filho teve.	1	2	3	4	5	6	7
… respiraçã o bucal por obstrução nasal?	1	2	3	4	5	6	7
… resfriados ou infecçõ es das vias aéreas superiores?	1	2	3	4	5	6	7
… secreçã o e congestão nasal?	1	2	3	4	5	6	7
… dificuldade em engolir alimentos?	1	2	3	4	5	6	7
Problemas emocionais							
Nas últimas 4 semanas, com que frequênciao seu filho teve…							
…alteraçõ es do humor ou acessos de raiva?	1	2	3	4	5	6	7
… comportamento agressivo ou hiperactivo?	1	2	3	4	5	6	7
… problemas disciplinares?	1	2	3	4	5	6	7
Problemas do quotidiano							
Nas últimas 4 semanas, com que frequência o seu filho teve…							
…sonolência diurna excessiva?	1	2	3	4	5	6	7
… episódios de falta de atençã o ou concentraçã o?	1	2	3	4	5	6	7
… dificuldade ao levantar da cama de manhã?	1	2	3	4	5	6	7
Opinião do Informante							
Nas últimas 4 semanas, com que frequência os problemas acima descritos…							
…causaram preocupação com a sua saúde?	1	2	3	4	5	6	7
… preocuparam-no pelo seu filho não poder respirar ar suficiente?	1	2	3	4	5	6	7
… interferiram com as suas actividades diárias?	1	2	3	4	5	6	7
… deixaram-no frustrado?	1	2	3	4	5	6	7

### Statistical analysis

Data was entered into a computer database developed specifically by the IT Department of the hospital. The data sets were processed with statistical analysis software SPSS (Statistical Package for the Social Sciences) version 19 (SPSS, Inc, USA).

Demographic and clinical data sets (age, gender, socioeconomic status according to Graffar^7^, level of education of caregivers, history of recurrent tonsillitis, Friedman^8^ classification to evaluate the position of the tongue and the degree of tonsillar hypertrophy, adenoid hypertrophy assessment) were reviewed in the statistical analysis.

The survey's degree of internal consistency (Cronbach's alpha) was validated based on the level of homogeneity seen between the different items of the questionnaire (adequate when > 0.70).

For quantitative variables, a correlation between each individual item and the total score of the OSA-pv-18 was assessed using the Pearson correlation coefficient. Convergent validity is verified when the Pearson correlation coefficient is greater than 0.20.

## RESULTS

The sample consisted of 51 Caucasian children, 35 males (69 %) and 16 females (31 %), with a mean age of 5 ± 2 years (2–11 years) with upper and lower quartiles at 4 and 6 years.

According to the Graffar socioeconomic classification, one child belonged to class I (2%), two to Class II (4%), 35 to Class III (69%), 13 to Class IV (25%), and none to Class V.

Two caregivers (4%) graduated from university, three (6%) graduated from high school or two-year degree programs, 11 (22%) did not complete high school or two-year degree programs, and 35 (69%) completed elementary school.

When history of recurrent tonsillitis was considered, 26 children (51%) met the Paradise^9^ criteria. On physical examination, 31 children (61%) had Friedman grades 3 or 4 tonsillar hypertrophy, while the remaining 20 (39%) had grade 2 tonsillar hypertrophy. The position of the tongue, according to Friedman's classification, was grade I in 18 children (35%) and grade II in the remaining 33 children (65%). Cephalometric evaluation was used to assess the degree of adenoid hypertrophy, which was determined by the ratio between the width of the adenoid tissue and the anteroposterior diameter of the nasopharynx after drawing a line tangential to the spheno-occipital synchondrosis intersecting the soft palate. Fifteen percent of the children had grade 2 adenoid hypertrophy (adenoid-nasopharynx ratios of 26% to 50%), 26% had grade 3 adenoid hypertrophy (ratios of 51% to 75%) and 59% had grade 4 adenoid hypertrophy (ratio > 75%).

Caregivers answered the surveys while at the hospital in the presence of a physician. The mean time to complete the survey was 7 minutes (4–13 minutes). Five participants (10%) found the questionnaire difficult to understand. On item ‘sleep disturbance’, the percentages of children who had a score of five or higher (meaning the symptom was present at least ‘a good bit of the time’) were: 76% for loud snoring; 41% for breath holding spells or pauses in breathing at night; 33% for choking or making gasping sounds while asleep; and 53% for restless sleep or frequent awakenings from sleep. On item ‘physical symptoms’, mouth breathing because of nasal obstruction was present at least ‘a good bit of the time’ in 37 children (73%); frequent colds or upper respiratory infections in 18 (35%); nasal discharge or a runny nose in 27 (53%); and difficulty in swallowing foods in 11 (22%). Emotional symptoms (items 9–11) and daytime function (items 12–14) were the two areas with the lowest mean scores. Approximately 59% of the caregivers felt concerned about the health of their children; 59% also felt concern that their children were not breathing enough air; 33% felt that these problems interfered with performance in daily activities; and 33% felt frustrated with the children's condition most of the time (Table 1).

**Table 1:** OSA-18-pv - Answers given to individual item answers for 51 children with OSAS (scale from one to seven, in which, 1) none of the time, 2) rarely any of the time, 3) a little of the time, 4) some of the time, 5) a good bit of the time, 6) most of the time, 7) all of the time).

Item	Subgroup	Content	Answers scored ≥ 5 (%)	Mean scores per group
1	Sleep disturbance	Loud snoring	39 (76)	
2	Sleep disturbance	Breath pauses	21 (41)	4.64
3	Sleep disturbance	Choking/gasping	17 (33)	
4	Sleep disturbance	Restless sleep	27 (53)	
5	Physical symptoms	Mouth breathing	37 (73)	
6	Physical symptoms	Colds/URTI	18 (35)	4.25
7	Physical symptoms	Rhinorrhea	27 (53)	
8	Physical symptoms	Dysphagia	11 (22)	
9	Emotional problems	Mood swings	11 (22)	
10	Emotional problems	Aggressive/hyperactive behavior	15 (29)	2.80
11	Emotional problems	Discipline problems	9 (18)	
12	Daytime function	Hypersomnia	8 (16)	
13	Daytime function	Attention deficit	12 (23)	2.88
14	Daytime function	Difficulty waking up	10 (20)	
15	Caregiver concern	Worry about the child's health	30 (59)	
16	Caregiver concern	Child is not getting enough air	30 (59)	4.18
17	Caregiver concern	Missing daily living activities	17 (33)	
18	Caregiver concern	Caretaker frustration	17 (33)	

Reliability analysis was carried out on SPSS based on internal consistency, yielding a Cronbach's alpha of 0.821 considering all items in the study. Converging validity was verified through the Pearson correlation coefficient, and each item was seen to have a statistically significant correlation with the OSA-18 total score and a Pearson coefficient correlation above 0.20 (Table 2).

**Table 2:** Pearson correlation coefficient between each item and total scores in the OSA-18-pv, and significance levels (n = 51).

Item	Content	Pearson correlation coefficient with OSA-18-pv scores	Statisticalsignificance (*P*)
1	Loud snoring	.528**	0.000
2	Breath pauses	.410**	0.001
3	Choking/gasping	.378**	0.003
4	Restless sleep	.454**	0.000
5	Mouth breathing	.452**	0.000
6	Frequentcolds/URTI	.498**	0.000
7	Rhinorrhea	.612**	0.000
8	Dysphagia	.405**	0.002
9	Mood swings	.623**	0.000
10	Aggressive/hyperactivebehavior	.607**	0.000
11	Discipline problems	.377**	0.003
12	Hypersomnia	.371**	0.004
13	Attention deficit	.318*	0.011
14	Difficulty waking up	.236*	0.048
15	Caretaker worried about child's health	.541**	0.000
16	Caretaker thinks child is not getting enough air	.695**	0.000
17	Missing daily living activities	.699**	0.000
18	Caretaker frustration	.704**	0.000

** Correlation is significant at a level of 0.01 (1-tailed).

* Correlation is significant at a level of 0.05 (1-tailed).

Based on the OSA-18-pv total score, the impact of OSAS on quality of life was mild in 14 cases (28%), moderate in 22 (43%), and severe in 15 (29%) (Table 3).

**Table 3:** The impact of OSAS on the quality of life of 51 children.

OSA-18-pv Total score	Impact on quality of life	Frequency (n)	Percent (%)
< 60	Mild	14	27.5
[60–80]	Moderate	22	43.1
≥ 80	Severe	15	29.4

## DISCUSSION

The OSA-18 is a quick, easy-to-use, highly reliable and consistent test used to evaluate the subjective aspects of quality of life in children with OSAS^6^. This study showed that the OSA-18-pv is an adequate translation of its original version in English, as demonstrated by the high reliability manifested through a Cronbach's alpha of 0.821 and significant consistency illustrated by the correlation between individual test items and total scores, which yielded a Pearson correlation coefficient > 0.20 and *p* < 0.05. Generally, the caregivers of the children with OSAS found the survey easy to understand; the five caregivers (9.8%) with medium-to-low income levels and low levels of education found the questions difficult to understand.

This survey allows physicians to better assess the impact of OSAS on affected children and their families, in addition to improving the selection from different treatment classes when combined with other clinical parameters and objectives. The survey is a quick test that can be used by physicians of various specialties.

The consistency and reliability of this study can be attributed to the fact that a guiding protocol was used and that the data sets were collected by the same author. Our protocol for Obstructive Sleep Apnea Syndrome^10^ includes systematic interviews in which caregivers are asked about the nocturnal signs and symptoms related to obstructive disease, including parasomnias, daytime symptoms, signs of adenotonsillar hypertrophy, and cognitive and behavioral problems. The second part of the protocol revolves around the application of the OSA-18-pv to caregivers in order to assess the impact of the disease on their lives. The children also undergo complete physical examination, which includes ENT evaluation, an analysis of their development in terms of height and body weight, and cardiovascular examination.

The most frequently reported symptoms in the OSA-18-pv survey were sleep disturbances (items 1–4), followed by physical symptoms (items 5–9), and caregiver concerns (items 16–18), as similarly reported by other authors^6^. Emotional problems and daytime function (items 10–15) were the areas with the lowest mean scores, although symptoms were often related to OSAS, according to the literature^11^, and interfered significantly with the quality of life of patients with OSAS. The OSA-18 also has the advantage of considering the neurobehavioral problems of children, while polysomnography does not take psychological components into account.

## CONCLUSION

The OSA-18-pv is a quick, easy-to-use instrument used to determine the quality of life of subjects with OSAS. The survey can be used in future research studies. More scientific research is needed in the area of pediatric OSAS to determine diagnostic criteria, correlate survey data with quality of life, clinical parameters with polysomnography results, to thus determine when it is necessary to carry out polygraphic sleep recordings/polysomnography to allow the timely referral of patients to ENT care and the identification of good candidates for surgery.
